# Dextran as a Resorbable Coating Material for Flexible Neural Probes

**DOI:** 10.3390/mi10010061

**Published:** 2019-01-17

**Authors:** Dries Kil, Marta Bovet Carmona, Frederik Ceyssens, Marjolijn Deprez, Luigi Brancato, Bart Nuttin, Detlef Balschun, Robert Puers

**Affiliations:** 1 ESAT-MICAS, KU Leuven, Kasteelpark Arenberg 10, 3001 Leuven, Belgium; fceyssen@esat.kuleuven.be (F.C.); luigi.brancato@esat.kuleuven.be (L.B.); puers@esat.kuleuven.be (R.P.); 2 Laboratory for Biological Psychology, Brain & Cognition, KU Leuven, Tiensestraat 102, 3000 Leuven, Belgium; marta.bovetcarmona@kuleuven.be (M.B.C.); detlef.balschun@kuleuven.be (D.B.); 3 Experimental Neurosurgery and Neuroanatomy, UZ Herestraat 49 box 7003, 3000 Leuven, Belgium; marjolijn.deprez@kuleuven.be (M.D.); bart.nuttin@uzleuven.be (B.N.)

**Keywords:** dextran, neural probe, microfabrication, foreign body reaction, immunohistochemistry, polymer, chronic

## Abstract

In the quest for chronically reliable and bio-tolerable brain interfaces there has been a steady evolution towards the use of highly flexible, polymer-based electrode arrays. The reduced mechanical mismatch between implant and brain tissue has shown to reduce the evoked immune response, which in turn has a positive effect on signal stability and noise. Unfortunately, the low stiffness of the implants also has practical repercussions, making surgical insertion extremely difficult. In this work we explore the use of dextran as a coating material that temporarily stiffens the implant, preventing buckling during insertion. The mechanical properties of dextran coated neural probes are characterized, as well as the different parameters which influence the dissolution rate. Tuning parameters, such as coating thickness and molecular weight of the used dextran, allows customization of the stiffness and dissolution time to precisely match the user’s needs. Finally, the immunological response to the coated electrodes was analyzed by performing a histological examination after four months of in vivo testing. The results indicated that a very limited amount of glial scar tissue was formed. Neurons have also infiltrated the area that was initially occupied by the dissolving dextran coating. There was no noticeable drop in neuron density around the site of implantation, confirming the suitability of the coating as a temporary aid during implantation of highly flexible polymer-based neural probes.

## 1. Introduction

Ever since Italian physician Luigi Galvani discovered that nerves and muscles were electrically excitable (1791) neural electrodes have proven to be an essential tool in neuroscience research as well as emerging clinical applications [1]. An example of such a development is the field of neuroprosthetics, which is concerned with the development and implementation of devices that can replace a motor, sensor or cognitive modality that might have been lost as a result of an injury or a disease [2]. The most well-known example is the cochlear implant which consists of an external unit that collects sound waves, processes them and in turn transfers the signals to the auditory nerve through a microelectrode array, substituting the function of the ear drum and stapes [3]. 

Things get more complex when we try to use this concept to replace lost brain function as is the case for, e.g., motor prosthetics [4]. Although penetrating microelectrodes that are suitable for this kind of application have undergone great improvements regarding the high density recording and stimulation of neuronal tissue, even at single-cell resolution, their limited chronic reliability is still the major limitation blocking widespread applicability [5]. Earlier experiments have shown that electrode performance typically starts to decrease a few weeks after implantation due to a progressive inflammatory tissue response, also known as the Foreign Body Reaction (FBR) [6,7,8]. The FBR causes the number of active channels to decrease, and the quality of the recorded signal to drop over time as scar tissue is formed around the implant, eventually leading to sensing inaccuracies, instability and failure of the implant. The FBR is considered the main reason behind the deterioration of microelectrode systems. The dynamic progression of the response results in instability in the recording quality over time, eventually leading to failure [9,10,11]. 

The trend mentioned above is recognized quite often in old as well as recent literature. Researchers describe a steady increase in impedance over the first four to six weeks after implantation which can be attributed to the expanding glial scar [12]. This is confirmed by the noticeable decrease in quality of the electrical recordings over time, as well as the post-mortem immunohistochemical analysis of the brain tissue [13]. From this information, it is evident that most of the problems related to chronic reliability can be traced back to the electrode design and its material properties, especially during the acute phase of the FBR. When comparing the characteristics of traditional materials for electrode fabrication to those of brain tissue, we immediately notice a large difference in Young’s modulus. Silicon and metals have moduli in the order of hundreds of GPa, while the stiffness of brain tissue is around 0.1 to 1.2 MPa [14]. After implantation, this mismatch will result in micromotions between the electrode and the tissue as the brain pulsates under influence of, e.g., blood flow, creating a constant source of irritation and thus a prolonged inflammatory reaction [5]. Several studies have examined the effect of implant stiffness on the surrounding brain tissue, providing evidence that using softer materials with a stiffness resembling that of the brain tissue reduces the aforementioned mismatch and attenuates the adverse foreign body reaction [15].

In general, it can be stated that the use of soft polymers increases the chronic reliability of penetrating microelectrodes. Practically however, this does come with some limitations. The most important one is the complexity of the surgical insertion of the probes, since many are too soft to penetrate the brain tissue without buckling, let alone puncture the dura. A frequently used solution is the use of an additional stiffening structure. Examples include stiff backbone layers [16], insertion shuttles [17] and biodegradable coatings [18]. These options however do come with some disadvantages. The stiff backbone layer is permanently attached to the implant and does not dissolve, limiting the flexibility of the device. Insertion shuttles on the other hand are quite bulky and temporarily increase the footprint of the implant, resulting in dimpling during insertion and additional, unnecessary damage to the neural tissue during implantation. The least invasive solution is to temporarily stiffen the implant using a bioresorbable coating, allowing the implant to regain its flexibility after the dissolution. Several types of bioresorbable materials have already been analyzed as potential candidates. Popular choices include silk fibroin [19], hydrogels, poly(lactic-co-glycolic acid) (PLGA), sucrose [20] and maltose [21]. Choosing the correct material for application is however no easy task, as each class of materials has its own (dis)advantages [22]. Sugar-based materials are typically characterized as materials with high Young’s moduli (35 GPa) and a fast resorption time. This allows the coated probe to regain its flexibility almost immediately after implantation. There is, however, a practical disadvantage. The user only has one chance for implantation, as the coating immediately softens upon contact with the cerebrospinal fluid (CSF). In an attempt to counter this problem, we research the use of dextran as a temporary coating material. Apart from its use in medicine as an antithrombic or volume expander for hypovolaemia [23], dextran not only limits non-specific cell adhesion [24] but as it is a complex branched glucan, its dissolution rate and mechanical stiffness can be tuned by varying the chain length of the molecule. First, a thorough characterization of the proposed material is executed in which the dissolution rate and mechanical properties are determined. Based on this analysis, the buckling force of a coated electrode can be calculated and compared to the force needed to puncture several anatomical regions of the brain. Afterwards, the dextran coated polymer probes are subjected to an in vivo experiment in which their performance is evaluated based on their ability to puncture the brain as well as the severity of the evoked FBR.

## 2. Materials and Methods 

### 2.1. Selection of Materials

As stated in the introduction, the use of soft polymer materials is critical for the development of reliable chronic neural probes. For this research, Parylene-C (Specialty Coating Systems) was chosen as the base material. Its dielectric properties, high strength under bending and biocompatibility make it an excellent candidate for our application. The flexibility of Parylene-C significantly increases the mechanical compliance between the device and the soft biological tissue surrounding it. The Young’s modulus of Parylene-C also closely resembles that of brain tissue. A unique feature of parylene-C is that it can be coated using chemical vapor deposition (CVD) in vacuum, which enables a conformal coating. Additionally, Parylene-C received United States Pharmacopeia (USP) Class VI and International Organization for Standardization (ISO) 10993 compliance, making it a Food and Drug Administration (FDA) approved material for implantation.

Platinum was chosen as the material of choice for the conductors due to its biocompatibility and inertness in a biological environment and high charge injection limit. 

Dextran (Sigma-Aldrich) was chosen as the coating material. Three types of dextran, with varying molecular weights, were analyzed: 40, 100, and 500 kDa. 

### 2.2. Electrode Design and Fabrication

**Figure 1:**
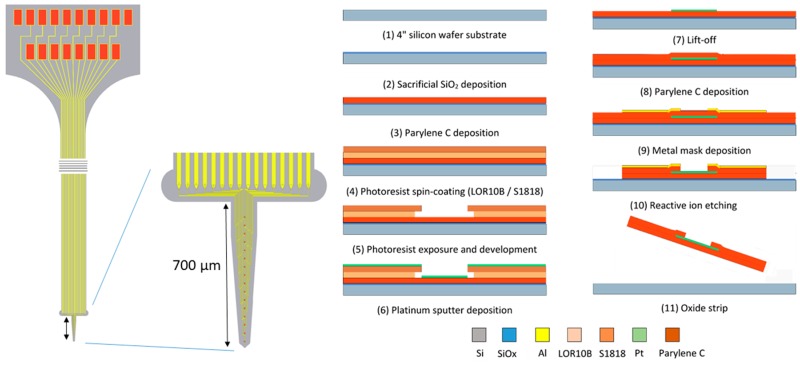
Electrode layout and microfabrication process.

All experiments were performed using a Parylene-C shank electrode with a tapered profile (Figure 1.). The probe width ranged from 72 µm at the tip to 218 µm at the base. The length of the shank was 700 µm, allowing it to penetrate all layers of the rat cortex after implantation. The microelectrode was fabricated using a standard two-mask, three-layer microfabrication process that was executed on a standard 4 inch silicon carrier wafer with a thickness of 500 µm. The following workflow was applied, as partially described in an earlier publication [25]:

- The 4 inch silicon carrier wafers were thoroughly cleaned using piranha etchant (4 H_2_SO_4_:1 H_2_O_2_) to remove any organic contaminants. Afterwards, a HF-dip (2% HF) was performed followed by a rinsing cycle in DI water. The substrate was dried using purified nitrogen.
- A 400 nm thin layer of silicon oxide was grown on the substrate using a wet thermal oxidation process. The oxide served as a sacrificial layer which aids in the final release of the devices from the carrier wafer in a later step.
- A first insulation layer of Parylene-C was deposited, yielding a 7 µm thick layer.
- The 400 nm thick Pt conductors were deposited by sputter coating on a lithographically patterned photoresist bilayer (LOR10B/S1818). The lift-off process was completed by soaking the wafers in n-methyl-2-pyrrolidone (NMP) overnight at room temperature.
- A second layer of Parylene-C, with a thickness of 7 µm, was deposited using a similar process.
- The device shape was defined by reactive ion etching (RIE) of the Parylene-C using an aluminum hard mask. The device outline was lithographically patterned using a negative photoresist on top of which a 40 nm thick layer of aluminum was thermally evaporated. After a lift-off step in acetone, the wafers were ready for RIE. As platinum is not etched by the RIE plasma, it also functions as an etch stop, which opens up the electrode contacts and bond pads.
- The wafer was annealed at a temperature of 200 °C for 4 h (2 °C/min ramping rate) in a nitrogen atmosphere to relax any residual stress that was built up during the processing, as well as to prevent delamination [26].
- Finally, the wafer was soaked in a 1% HF solution that removed the Al hard mask and underetched the sacrificial silicon oxide layer. After 1 h of etching, the adhesion between the Parylene-C electrode and the carrier wafer was reduced to such a level that the electrodes could be peeled off the carrier wafer using tweezers.

After completing the fabrication process the implants were stored in 70% ethanol for disinfection purposes. 

To embed the electrode array, the neural probe was dipcoated in a highly viscous solution of molten dextran. Initially the dextran powder was dissolved in deionized water (50 wt %) before placement on a hotplate at 200 °C. After the evaporation of the water that was present in the solution, the dextran started to melt and take on a liquid state. The neural probe was fixed to a motorized z-stage and submerged in the solution. Afterwards, the probe was drawn from the solution, coating it with a layer of adhesive dextran, which immediately crystallized under influence of the lower room temperature.

**Figure 2:**
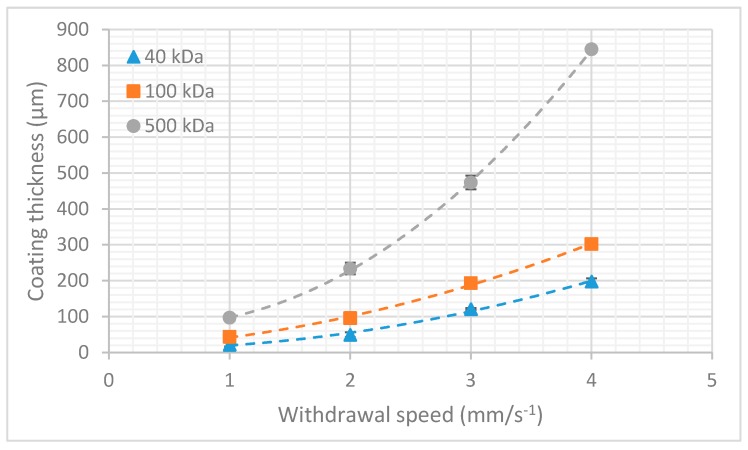
Coating thickness vs. withdrawal speed in function of molecular weight. The error bars represent the standard deviation (*n* = 6).

The final film thickness was determined by the interplay between the entraining and draining forces acting on the film. This can be theoretically described by the Landau–Levich equation and the capillary regime equation which are a function of both the viscosity of the solution and the withdrawal speed of the probe. In order to characterize the aforementioned dipcoating process, an experiment was performed to determine the relationship between film thickness and withdrawal speed for dextran of different molecular weights (Figure 2). Six samples were tested for each combination of withdrawal speed and molecular weight. The plotted coating thickness consists of the combined thickness of dextran deposited on both sides of the shank. 

As expected from the theoretical approximation, a higher molecular weight dextran (higher viscosity) resulted in a higher film thickness. For more information on the physical principles behind the dipcoating process, I refer to the review article by Rio and Boulogne [27].

### 2.3. Micromechanical Characterization

In order to achieve successful surgical placement of neural probes, the main requirement is that the probes are mechanically robust enough to penetrate the brain. Ideally, the buckling force threshold of the neural probe must be higher than the penetration force needed for implantation. As in almost all cases, the thin polymer-based implants are too flexible for implantation and some sort of mechanical reinforcement is needed to temporarily increase the probe stiffness. Ideally, this mechanical augmentation is also temporary, allowing the electrode to regain its flexibility once it is implanted. Setting a fixed number on the amount of force needed to penetrate brain tissue is however very difficult, as the mechanical properties of brain tissue are highly dependent on a multitude of factors, such as the species of the subject animal, its age or the stiffness characteristics of the functional zone in the brain that is targeted. All of these tissue-specific variables are critical in determining the penetration force of neural probes for the application of interest [28].

For our intended application, which is a chronic implantation through the dura in the underlying sensorimotor cortex, we need to penetrate the stiffest part of the brain, the dura mater. The dura is the outermost meningeal covering of the brain and is made out of dense connective tissue. It has a Young’s modulus that is several orders of magnitude higher than the cortex, cerebellum or the hippocampus. Many research groups prefer to remove the dura using sharp forceps to increase the ease of insertion of flexible neural probes. Performing a durotomy is however never without risk and should be avoided when trying to maximize the chronic reliability of the probe. 

Based on earlier experiments reported in literature, we can make a rough estimation of the penetration force that is needed. A study by Jensen et al., which used silicon probes to penetrate rat cerebral cortex, concluded that penetration forces are in the range of 0.45 to 1.15 mN with an average of 0.775 mN [29]. Values in the range of 0.54 to 2.48 mN with a 1.25 mN average are reported by Sharp et al. in the case of individual stainless steel probes [30]. The small differences in penetration force ranges can be attributed to the difference in tip shape, as Sharp explored insertion of blunt implants, whereas Jensen focused on very sharp probe tips. Based on the information available in literature, we can make a safe estimation that individual tapered probes should be able to withstand a force of 1.5 mN to penetrate the rat cortex. This value can now be used as a design goal for the required buckling force threshold of neural probe.

By modeling penetrating neural probes as beams that are clamped at one end and pinned at the other, it is possible to make a mathematical estimation of the buckling force threshold of flexible probes. This simplification results in quite accurate results as the probes are fixed at their base since they are reversibly adhered to an insertion platform/stereotactic frame during implantation. They are considered pinned at the other end from the moment the tip contacts the dura. We can use Euler’s formula to determine the critical load at which buckling occurs.

(1) $$Pcr=\frac{\left(K\cdot pi^{2}\cdot E\cdot I\right)}{L^{2}},$$

(2) $$I=\frac{1}{12}\cdot b\cdot h^{3},$$

where *Pcr* = buckling force threshold, *K* = column effective length factor = 2.045, *E* = elasticity modulus, *I* = area moment of inertia, *L* = unsupported beam length, *b* = beam width, *h* = column thickness.

As an example, we can take a look at the critical load of our untreated Parylene-C shank electrode. The unsupported length of the electrode during implantation is 1 mm with a width of 190 µm and a thickness of 14 µm. The elasticity modulus of Parylene C is 2.8 GPa.

Theoretical analysis results in a buckling force threshold of 2.4 mN, indicating that the electrode is strong enough to withstand penetration in brain tissue without buckling. If we want to puncture the dura however, the electrode will need to be able to withstand a critical load of around 10 mN, which we would not be able to achieve with the electrode in its current form. This problem can be circumvented by fabricating a thicker electrode, but this will increase the stiffness of the implant and have an adverse effect on the ‘mechanical match’ of the electrode with the brain tissue, which is a critical requirement to improve long-term reliability of the implants. As stated earlier, the solution proposed in this paper is the use of a thin dextran coating that will temporarily stiffen the electrode.

To assess the level of mechanical reinforcement that can be obtained by a dextran coating, a set of micromechanical experiments was performed. Uncoated probes were taken as a reference.

**Figure 3:**
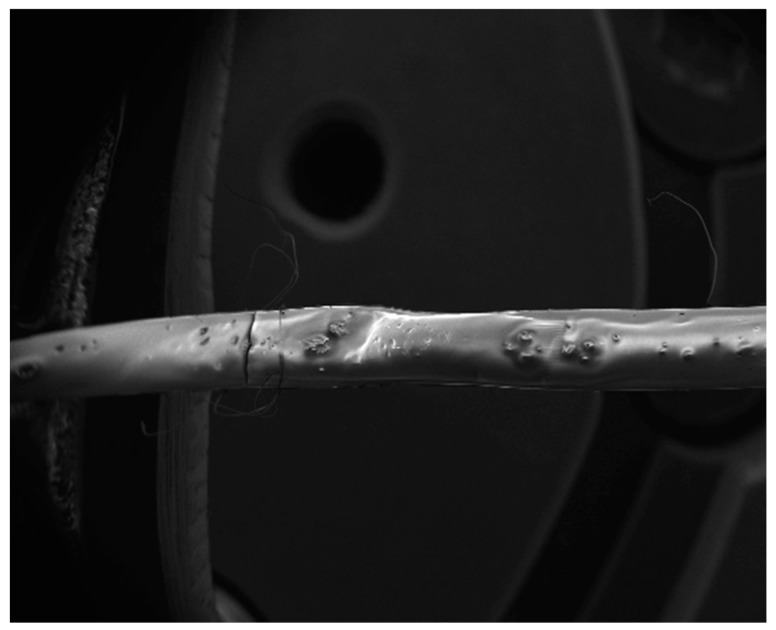
Electron micrograph of a dextran coated Parylene-C neural probe. A crack can be seen on the left-hand side of the probe which was formed after buckling. Reproduced with permission from [31]; published by IOPscience, 2017.

**Figure 4:**
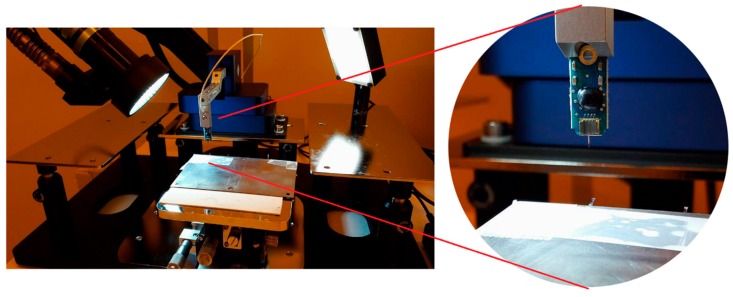
Test setup for measuring bending stiffness using the Femtotools FTA-M02 micromechanical testing system. Close-up shows the vertical indenter probe with integrated force sensor. Reproduced with permission from [31]; published by IOPscience, 2017.

The samples were fixed on a substrate with a known part of the shank extending over the edge. The floating part of the shank was displaced using a Femtotools FTA-M02 micromechanical testing system (Figure 3 and Figure 4). Force measurements were taken during the displacement and the bending stiffness of the samples was calculated. The Young’s modulus of the samples was approximated using the formula for bending of a beam under a point load:

(3) $$E=\frac{k\cdot L^{3}}{3\cdot I}.$$

With *I* the area moment of inertia:

(4) $$I=\frac{b\cdot h^{3}}{12}.$$

In these formulas, *L* represents the distance between the hinging point and the contact point of the indenter tip, *b* is the measured width of the sample and *h* is the sample thickness. The stiffness of the coating is orders of magnitude higher than the stiffness of the uncoated implant, allowing us to simplify the implant to a solid dextran beam model during mechanical characterization. 

### 2.4. Dissolution Rate

The dissolution rate of the coatings was determined in function of the molecular weight of the used dextran. Dextran slabs were prepared using a 1 cm × 1 cm × 1 cm mold, weighed and submerged in deionized water for a known amount of time. At regular time intervals, the samples were removed from the container, allowed to dry in an oven at slightly elevated temperature (40 °C), and weighed again. This provides an indication of the amount of dextran that dissolves over time. The dextran slabs were submerged while still in the mold to keep the surface area exposed to the dissolution rate constant in time, which results in a more accurate representation of the dissolution rate. 

### 2.5. In Vivo Experiment

All experiments were performed in accordance with the Belgian and European laws, guidelines and policies for animal experimentation, housing and care (Belgian Royal Decree of 29 May and European Directive 2010/63/EU on the protection of animals used for scientific purposes of 20 October 2010). The animal experiments were performed on six male Wistar rats, weighing about 250 g on arrival. The animals were housed and maintained in standard cages under conventional laboratory conditions with ad libitum access to food and water and a 12 h light/12 h dark cycle. All experiments were conducted during the light phase of the animal’s activity cycles. 

#### 2.5.1. Implantation Procedure

Prior to the surgical procedure the animals were anesthetized using an isoflurane/oxygen pump system (Iso-vet Surgivet at 3% isoflurane for induction and 1% to 2% for maintenance; O_2_ 1 L/min) after which they were placed in a stereotactic frame where constant isoflurane/oxygen was administered through a face mask. After removing the hair on the head, additional subcutaneous anesthetic (Xylocaine 2%, 1:200000 adrenaline, AstraZeneca) was applied. Then, the skin was opened and the skull was cleaned. Two holes were drilled, 4 mm distally from bregma, over the S1 sensorimotor cortex. First, the connector assembly was fixed on the skull together with a set of stainless steel screws that provide additional support. Afterwards, the electrode tip was inserted through the dura to a depth of roughly 700 µm and fixed in place using a drop of UV-cured dental cement (Tetric EvoFlow, Ivoclar Vivadent). Finally, the wound was sutured and pain relief was ensured for 24 h by administering 0.06 mg/kg of Vetergesic (Ecu phar). The implanted electrodes were temporarily stiffened using a 40 ± 7 µm thick dextran coating with a molecular weight of 40 kDa.

#### 2.5.2. Perfusion

Four months after implantation the experiment was stopped. The animals were sacrificed by administering a lethal dose of pentobarbital. Afterwards, an intracardial perfusion was executed to fixate the brain tissue. First the animals were perfused with 250 mL of PBS followed by 200 mL of 4% PFA. After extraction of the brain, it was stored in 4% PFA for 24 h before rinsing and storing in a solution of 20% sucrose and 0.1% sodium aziide in preparation for histology. Prior to slicing, the tissue was embedded in a 4% agar gel. Coronal slices with a thickness of 80 µm were taken using a Leica VT1000S vibrotome (Leica Biosystems Inc., Buffalo Grove, IL, USA). It should be noted that due to the fixation of the tissue a small amount of shrinkage occurred, resulting a minor error during the subsequent analysis [32].

#### 2.5.3. Histology

Before analysis the samples were stained for glial fibrillary acidic protein (GFAP) and neuronal nuclei (NeuN) which are standards for visualization of reactive astrocytes and neurons, respectively. The following procedure was performed:

- Overnight soak in blocking buffer (1% BSA, 0.1% Triton X100 in PBS) to reduce non-specific background staining
- Application of primary antibodies (1:100 MAB377 mouse anti-NeuN; 1:500 polyclonal rabbit anti-GFAP Z0334 in blocking buffer)
- Rinse in PBS (three times)
- 2 h incubation in blocking buffer
- Application of secondary antibodies (ALEXA fluor 488 (Abcam, Cambridge, UK) donkey anti-mouse IgG (H + L) and Cy3 Donkey Anti Rabbit IgG (H + L), 1:1000 in blocking buffer).
- Rinse in PBS (three times)

Subsequently, the samples were mounted on Superfrost microscope glasses using Sigma Fluoroshield. Imaging was done using a Leica TCS SP8 confocal microscope (Leica Microsystems, Wetzlar, Germany) using a 20× immersion objective (1024 × 1024 pixel imaging, 5% 488 nm laser power, 3% 552 nm laser power, Cy3 and ALEXA 488 filters and 950 and 830 amplifier gain, respectively). The analysis of the images was done using ImageJ image processing software. To quantify the amount of gliosis, the average thickness of the scar tissue was measured. Additionally, any change in neuronal density around the site of implantation was determined by counting neuronal cell bodies in concentric areas around the site of implantation. 

## 3. Results

### 3.1. Micromechanical Characterization

In order to make an accurate comparison with the clamped beam model, which is proposed in Section 2.3., rectangular Parylene-C dummy probes were used for the micromechanical characterization. Measurements were performed on three groups (40, 100, and 500 kDa) of six samples each. The uncoated implants had an average thickness of 14 ± 1 µm and an average width of 500 ± 3 µm. The coated samples had an average thickness of 163 ± 21 µm and an average width of 641 ± 15 µm. By varying the withdrawal speed during dipcoating, we were able to achieve the same coating thickness for all dextran types. This allows us to easily detect any changes in mechanical properties that are related to the molecular weight of the used dextran. Micromechanical testing reveals an average bending stiffness of 0.029 ± 0.005 N∙m^−1^ for the uncoated implants. Coating increased this average bending stiffness 360-fold to 10.5 ± 5.5 N∙m^−^^1^. The Young’s modulus of all coated samples is measured to be only 0.6 ± 0.1 GPa, proving that the enhanced structural stiffness can be mainly attributed to the thickness of the coating and not to the molecular weight of the dextran. Based on the data gathered during the experiment, we can determine, by using Equations (1) and (2), that a dextran thickness of 37 µm is required to meet the critical load of 10 mN that is required for penetration of the dura mater.

### 3.2. Dextran Dissolution Rate

**Figure 5:**
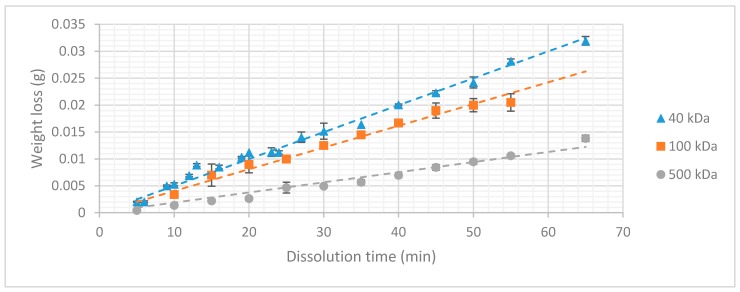
Weight loss in function of dissolution time (*n* = 6).

The results of the dissolution experiment are depicted in Figure 5. It is clearly visible that the higher the molecular weight of the dextran, the lower the weight loss over time becomes.

**Figure 6:**
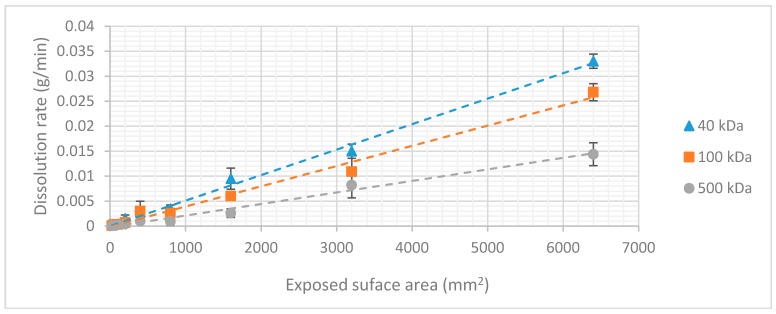
Dissolution rate in function of surface area exposed to the dissolution medium. The error bars represent the standard deviation (*n* = 3).

The aforementioned conclusions indicate a strong dependency between dissolution rate and molecular chain length that can be explained by the physical mechanism of sugar dissolution in water. When the sugar crystal comes into contact with water, the polar bonds of the water molecules form their own dipole-dipole bonds with the sugar molecules. These bonds are stronger than the intermolecular Vanderwaals forces which are present between the sugar molecules themselves, causing them to separate and bond to the water molecules. For molecules with high molecular weight (longer chain length) the formation of dipole-dipole interactions with the water molecules is more difficult. The surface area of the molecule exposed to the solute is relatively small compared to its size. This effect increases with the molecular weight, decreasing the dissolution rate. This allows tuning of the dissolution rate of dextran coatings by varying the molecular weight of the dextran. It is important to note that these three types of dextran can only be compared because the surface area exposed to the dissolution experiment was kept constant during the whole experiment. If the surface area is increased under constant volume, more dextran molecules would come into contact with the medium, increasing the rate at which the coating dissolves. This linear relationship was validated by an additional experiment in which the surface area exposed to the dissolution medium was varied by changing the dimensions of the dextran slabs (Figure 6).

The dissolution mechanism can be considered as a ‘layer-by-layer’ process. The thickness of the coating has no influence on the dissolution rate. The total dissolution time is determined by the volume of the coating. As we now have information on all the factors influencing the dissolution time of a dextran coating, a formula can be presented, so that the dissolution time of an arbitrary dextran coating can be predicted. The density of the coating is dependent on the molecular weight of the dextran used and has already been published in earlier work [31]. 

Combining the results of the experiments, it is possible to create a formula that can be used by the reader to either determine the dissolution rate of a dextran coating with a known surface area to volume ratio or to determine the specifics of a coating with a desirable dissolution time.

(5) $$\mathrm{Dissolution}\;\mathrm{time}=\frac{\left(\mathrm{Surface}\;\mathrm{area}*\mathrm{coating}\;\mathrm{thickness}\right)*\rho }{K\left(S,Mw\right)},$$

(6) $$\mathrm{coating}\;\mathrm{thickness}=\frac{\left(\mathrm{Dissolution}\;\mathrm{time}*K\left(S,Mw\right)\right)}{\mathrm{Surface}\;\mathrm{area}*\rho },$$

with *K(S, Mw)* being the dissolution rate in function of surface area and molecular weight of the used type of dextran.

This dissolution rate can be determined from Figure 5 in function of the surface area exposed to the dissolution medium and the molecular weight of the dextran. Using this formula, we now hold the tools to calculate the dissolution time of the probe we designed in the earlier sections The aforementioned layer-by-layer mechanism was verified by measuring the dissolution time of a known dextran coating and comparing it to its calculated value which was extracted from the “macro-model.” A dextran coating with a thickness of 37 µm (MW = 40 kDa) resulted in a dissolution time of roughly 250 s. We have to take into account that this is the dissolution time when the sample is submerged in a large volume of water, meaning that the concentration gradient in the system can be considered to be maximal during the whole dissolution process. 

### 3.3. Image Analysis

**Figure 7:**
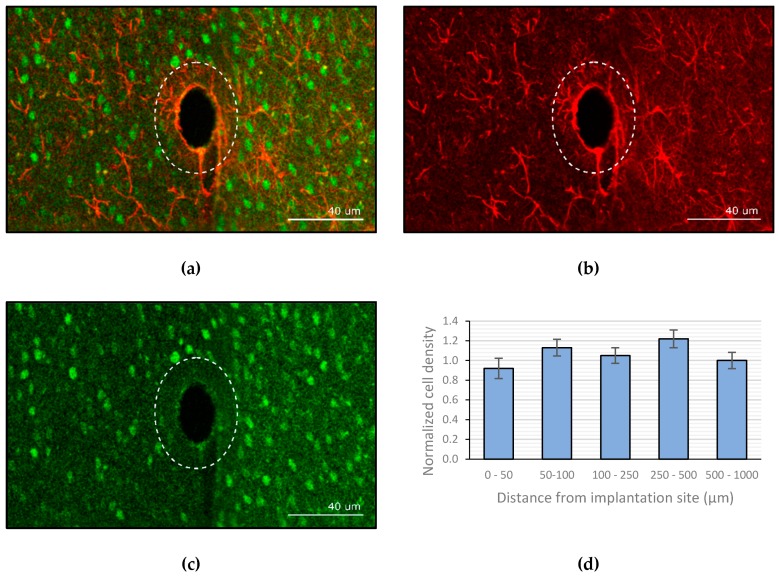
Confocal imaging of the brain slices (**a**) overlay of both the glial fibrillary acidic protein (GFAP) and neuronal nuclei (NeuN) stained channels. (**b**) GFAP channel. (**c**) NeuN channel. (**d**) Normalized neuronal density relative to site of implantation (the error bars represent the standard deviation, *n* = 6).

Analysis of the images shows the ‘neuronless’ zone in which the probe resided. The probe is no longer visible as it was removed from the brain during the brainectomy. This area has the same size as the cross section of the uncoated electrode, indicating that the active neurons have penetrated the area that was previously occupied by the dextran coating. As a reference, the outline of the coated electrode is visualized in Figure 7 by the dotted line. The implanted probe was coated with a ±40 µm thick layer of dextran (40 kDa). The amount of gliosis was quantified using the GFAP stained slices (Figure 7b) and revealed an average thickness of 3.05 ± 0.73 µm. Counting of viable neurons in the vicinity of the implant showed that there was no significant decline in neuron density when approaching the implant. Figure 7 shows the average neuronal cell density at several distances from the site of implantation, normalized to the density in the outer concentric circle. 

## 4. Discussion

The described experiments were executed to solve the practical problems that are associated with the use of highly flexible polymer-based neural probes. The goal we wanted to achieve was to find a coating material that could temporarily stiffen flexible probes, without evoking any additional effects concerning the FBR. 

We started by going back to the origin of the problem, a simplified mechanical model was used to describe the buckling behavior of a bare Parylene-C electrode. Based on the results, it became evident that additional reinforcement is necessary if we aim to puncture the dura without buckling of the probe. In the quest to find a suitable material, dextran came forward as an ideal candidate as its chemical composition (polysaccharide) allowed tuning of different parameters. After performing a micromechanical experiment, we were able to deduce that a dextran coating of 37 µm increases the critical load of the assembly sufficiently to prevent buckling of the presented neural probe. Additionally, the dissolution rate of the coating can be controlled by varying the molecular weight and thickness of the used dextran. Practically, this means that a fast dissolution time allows the electrode to regain its highly flexible state after minutes, preventing micromotions in an early stage, and thus strongly limiting the chronic FBR and the associated glial scar formation. These hypotheses were analyzed by performing a long-term in vivo experiment. Not only did the results show that the glial sheath only had a thickness of roughly 3 µm, but also showed that the area initially taken up by the dextran coating was re-occupied with active neurons, which is most likely related to the quick dissolution of the coating. Moreover, there was no noticeable decrease in neuronal density in the vicinity of the implant.

With chronic applicability in mind, this may be a very important characteristic of the dextran coating. The functionality of neuroprosthetic devices depends on their capability to form electrical connections with nearby neuronal dendrites and axons, and the glial sheath prevents regrowing neuronal processes from contacting the implant. It is therefore important that this sheath is as thin as possible, as a smaller distance between the recording electrode and the neuron(s) of interest minimizes this electrically resistive barrier and has a positive effect on signal quality. Ideally, we would like to evolve to the use of ultra-thin polymer-based implants with thicknesses in the range of several micrometers or lower. Recent literature proved that such devices can be implanted, reliably, for up to a year without inducing the formation of any scar tissue [33,34,35]. Dextran would be a perfect temporary coating material for reliably implanting this type of neural probes.

Compared to the other available bioresorbable materials which are often used for the same application, dextran behaves quite well. The higher Young’s modulus allows for a thinner coating, which leads to a smaller insertion footprint and dimpling effect. Especially, when the user aims to implant deep into the brain, a high Young’s modulus coating is beneficial. Additionally, there are the practicalities related to the surgical implantation. Fast-resorbing polymers such as maltose only offer a single chance of implantation, as the coating softens immediately upon contact with the CSF [21]. This effect can be countered by dextran as it is available with chain lengths ranging from 3 to 2000 kDa. The disentanglement of large, high molecular weight, molecules from the particle surface and subsequent diffusion to the bulk solution takes a longer time, resulting in a slower dissolution rate.

In the literature, we can find several more examples of studies in which a resorbable biomaterial was used to temporarily stiffen flexible neural probes. An article by Lind et al. describes the use of a gelatin to embed thin, flexible microelectrodes [36]. In vivo experiments showed that the body was able to completely eliminate the gelatin, without forming a permanent scar. The neuronal cell density in the immediate vicinity of the implantation site was only slightly reduced, with viable neurons still remaining in the examined region of interest. The effect of the swelling of gelatin is however not described and might result in additional compressive stress on the surrounding cells. Lecomte et al. suggest the use of Polyethylene glycol (PEG) or silk as a coating material [37]. Despite its high Young’s modulus, silk fibroin is not commercially available and tedious to process, making dextran a more interesting material. The alternative presented in the paper is the use of PEG, which dissolves within seconds after contact with cerebrospinal fluid, resulting in the practical problems that are mentioned before.

Overall, we can state that the conducted research proved that dextran is a practical and interesting coating material to stiffen flexible neural probes during the implantation phase. Future research will involve the use of dextran as a temporary coating material for an ultra-thin microelectrode array. 

## 5. Conclusions

In this paper we presented and evaluated a novel coating method for flexible implants to improve the chronic reliability of neural probes, which is mainly afflicted by the FBR. By adjusting the probe stiffness to mimic that of brain tissue, this inflammatory reaction can be minimized, resulting in an increased lifetime. Using thin, flexible polymers as a base material solves this problem, but comes with its own limitations. To prevent buckling during surgical insertion the polymer probe needs to be stiffened temporarily. For this purpose, a thin dextran coating is applied which increases the critical load of the assembly. Additionally, the resulting stiffness and dissolution rate of the dextran coating can be tuned to the requirements of the user. All allegations were tested in a long-term in vivo experiment. After four months of electrode implantation only a small lesion remained, enveloped by scar tissue with an average thickness of 3 µm. Apart from a very small glial scar, no drop in neural density was observed, therefore proving that the presented technology allows successful implantation of highly flexible polymer-based neural probes without evoking a severe immune response.
